# Experimentation with tobacco during adolescence as a factor influencing treatment of smoking in adulthood. A retrospective cohort

**DOI:** 10.1590/1516-3180.2018.0504140319

**Published:** 2019-07-29

**Authors:** Carolina Hanna Chaim, Erica Rosanna Siu, Carlos Felipe Cavalcanti Carvalho, Fernanda Piotto Frallonardo, Flavia Ismael, Arthur Guerra de Andrade, Antonio Ventriglio, Julio Torales, Dinesh Bhugra, João Mauricio Castaldelli-Maia

**Affiliations:** I MD. Research Associate, Department of Psychiatry, Faculdade de Medicina da Universidade de São Paulo (FMUSP), São Paulo (SP), Brazil.; II PhD. Research Associate, Department of Psychiatry, Faculdade de Medicina da Universidade de São Paulo (FMUSP), São Paulo (SP), Brazil.; III MD. Research Associate, ABC Center for Mental Health Studies, Santo André (SP), Brazil.; IV MD. Research Associate, ABC Center for Mental Health Studies, Department of Neuroscience, Faculdade de Medicina do ABC (FMABC), Fundação ABC, Santo André (SP), Brazil.; V MD, PhD. Vice-President, ABC Center for Mental Health Studies, Department of Neuroscience, Faculdade de Medicina do ABC (FMABC), Fundação ABC, Santo André (SP), Brazil. Auxiliary Professor of Medicine, Universidade de São Caetano do Sul (USCS), São Caetano do Sul (SP), Brazil.; VI MD, PhD. Associate Professor, Department of Psychiatry, Faculdade de Medicina da Universidade de São Paulo (FMUSP), São Paulo (SP), and Full Professor, ABC Center for Mental Health Studies, Department of Neuroscience, Faculdade de Medicina do ABC (FMABC), Fundação ABC, Santo André (SP), Brazil.; VII MD, PhD. Honorary Researcher, Department of Clinical and Experimental Medicine, University of Foggia, Foggia, Italy.; VIII MD, PhD. Assistant Professor, Department of Psychiatry, Medical School, National University of Asunción, San Lorenzo, Central, Paraguay.; IX MA, MSc, MBBS, FRCP, FRCPsych, FFPH, PhD, FACP, FAMS. Emeritus Professor, Institute of Psychiatry, Psychology & Neuroscience, King’s College, London, United Kingdom.; X MD, PhD. Research Associate, Department of Psychiatry, Faculdade de Medicina da Universidade de São Paulo (FMUSP), São Paulo (SP), and Auxiliary Professor, ABC Center for Mental Health Studies, Department of Neuroscience, Faculdade de Medicina do ABC (FMABC), Fundação ABC, Santo André (SP), Brazil.

**Keywords:** Adolescent, Tobacco, Nicotine, Treatment outcome

## Abstract

**BACKGROUND:**

There are still few studies on predictors of smoking cessation in Brazilian samples. Experimentation with tobacco during adolescence (ETA) may be one of the important predictors.

**OBJECTIVE:**

This study aimed, within the context of a treatment-seeking group of subjects, to test the hypothesis that ETA negatively affects the outcome of smoking cessation during adulthood.

**DESIGN AND SETTING:**

Retrospective (historic) cohort study conducted at a psychosocial care center in São Paulo, Brazil, between 2007 and 2010.

**METHODS:**

Data on sociodemographics, smoking and medical profiles were obtained through self-report questionnaires that were completed at the baseline and at any follow-up appointment. Logistic regression models were constructed to describe factors associated with the outcome of smoking cessation, measured according to the self-reported four-week success rate among 367 outpatient smokers.

**RESULTS:**

ETA was found to be associated with not quitting smoking through the treatment (odds ratio = 0.57; 95% confidence interval = 0.33-0.96; P < 0.05), even after adjustment for dependence level, sociodemographics, nicotine patch use and number of years of smoking.

**CONCLUSIONS:**

Early exposure to nicotine may lead to higher risk of continuing smoking after treatment, in adulthood.

## INTRODUCTION

Cigarette smoking, including secondhand smoking, has been identified as the second leading risk factor regarding overall disease burden, only behind high blood pressure. Efforts have been made to control tobacco consumption since the 1970s, such that the overall estimated age-standardized prevalence of daily tobacco smoking declined by 25% for men and 42% for women between 1980 and 2012 globally. On the other hand, because of population growth, the number of smokers increased significantly worldwide, from 721 million in 1980 to 967 million in 2012.^1^ A Brazilian study^2^ documented a steep decline in smoking prevalence from 15.6% in 2006 to 10.8% in 2014. Despite this public health success story of reducing smoking, tobacco use continues to adversely influence global health patterns, leading to 5.7 million deaths, and 5.5% of disability-adjusted life-years (DALYs) in 2010.^3^^,^^4^

The health benefits of smoking cessation are well established and it is also known that Brazil presents a relatively high quit ratio, compared with other countries (around 47% of former daily smokers among those who were ever daily smokers).^5^ Considering this tendency among Brazilian smokers for them to seek to abstain from tobacco use, there is an opportunity to improve existing prevention and treatment strategies that can be seized. It is also known that a majority of smokers would like to stop and indeed attempt to stop many times. However, even though 60%-70% of smokers may intend to stop in any given year, only 3%-5% of them remain abstinent through an unassisted attempt, 7%-16% through receiving behavioral therapy and 24% through receiving behavioral therapy combined with pharmacotherapy.^6^^,^^7^

Understanding the determinants of stopping smoking is important for better defining the profile of patients seeking treatment and individuals needing to receive targeted preventive interventions. Nonetheless, there are still few studies on predictors of smoking cessation in the general population. A large systematic review found that only the dependence levels were consistently predictive of the success of these attempts.^8^ On the other hand, many studies have tried to elucidate the impact that other factors, such as nicotine replacement therapy, neuropsychiatric comorbidities, smoking more often during the first hours of wakefulness, abstinence symptoms, duration of treatment and early initiation of smoking, have on treatment outcomes.^9^^,^^10^^,^^11^ A pre-post field trial study tried to elucidate how an early start to tobacco use impacts treatment outcome and found that there was a positive association between the mean age at which smoking started and cessation rates. This showed that treatment was more likely to provide success when the mean age at which smoking started was greater.^12^

Adolescence is widely recognized as a time of greater risk-taking, compared with other age groups. Adolescents are more likely than other developmental age groups to start smoking cigarettes because of normative developmental processes, including heightened reactivity to novel and potentially rewarding stimuli and protracted maturation of cognitive control, along with lower heed given to guidance about the future and heightened sensitivity to peers.^13^ Exposure to nicotine among adolescents has been directly associated with greater risk of later nicotine dependence,^13^ although there is still discussion regarding the evidence of causal effects, as observed in animal models of adolescents, which are more sensitive to nicotine reward effects and less sensitive to their aversive effects.^13^

However, researchers have also considered the presence of shared genetic and environmental risk factors for both dependence and early age of onset. Regarding this issue, a monozygotic co-twin control study^14^ (controlled for genetic and familial-environmental effects) suggested that early nicotine exposure directly increased the level of later nicotine dependence and craving for cigarettes when unable to smoke, thus supporting the causal effect hypothesis. If the causal effect is true, then reducing early exposure should reduce the risk of subsequent nicotine dependence and all the implications and other disorders to which it predisposes.

## OBJECTIVE

The main objective of the present study was, in the context of a treatment-seeking group of subjects, to test the hypothesis that experimentation with tobacco during adolescence (ETA) is associated with not quitting smoking through treatment, in adulthood, by comparing the smoking cessation rates between two groups of individuals who completed a smoking treatment protocol (one group with and the other without ETA) and adjusting the results for 12 covariates, including the number of years of tobacco smoking, since this may be an important confounder.

## METHODS

### Design, ethics and setting

We conducted a six-week retrospective (historic) cohort study on all the patients who were being treated through a smoking cessation protocol at the Psychosocial Care Center for Alcohol and Drugs in the city of São Caetano do Sul, São Paulo, Brazil, from April 2007 to April 2010. Ethics approval was obtained from the local Institutional Review Board (April 13, 2011; no. 028/2011).

To calculate the sample size, we used the average success rate from smoking cessation treatment that was found among individuals using pharmacotherapy at week 12 in a previous important study (which was around 30%).^19^ Considering an alpha of 5% and an error of 10%, we found that we would need 80.6 individuals enrolled in the exposure subgroups. Fortunately, we had more than 81 individuals in both exposure subgroups (ETA and non-ETA).

### Participants

The subjects were recruited between July 2007 and December 2010 and data were collected using questionnaires applied at the baseline and using a follow-up chart (from T0 to T4; see section 2.1). Patients were recruited through banners that were displayed in the Psychosocial Care Unit (CAPS), and general healthcare professionals in the region were also asked to disseminate the anti-smoking program among smokers in the region. The mean age of the subjects was 51 years. Self-report questionnaires were completed at the baseline with the assistance of healthcare professionals, and evaluations and ratings were recorded at any follow-up appointment.

### Treatment protocol

The treatment protocol included six sessions of weekly group therapy and four consultations with a psychiatrist (T1 = 0; T2 = 1 week; T3 = 3 weeks; T4 = 6 weeks).^15^^,^^16^^,^^17^^,^^18^ The group therapy sessions were composed of up to 15 people and were based on the principles of cognitive-behavioral psychotherapy.^2^

### Measurements

In the present study, the cutoff age for starting to use tobacco was taken to be 19 years of age, based on the cutoff used in several recent studies among adolescents^20^ and following data on the age of initiation among Brazilians,^2^ in order to establish the measurement for exposure to tobacco (experimentation with tobacco during adolescence, ETA).

The outcome measurement of success was considered to be four weeks of self-reported abstinence, in accordance with the criteria for “self-reported four-week quitter” that are encompassed in the Russell standard.^21^ Individuals were deemed to have been unsuccessful both if they had not achieved success by the end of the protocol period and if they dropped out.

### Statistical analysis

The Stata software, version 11, was used to create the database of information from the questionnaires and protocols and to analyze the data. Using the “enter” method, univariate logistic regression and multiple logistic regression were performed. Descriptive analysis comparing ETA and non-ETA groups regarding their sociodemographic and smoking profile was carried out. Then, twelve variables were included in the multiple logistic regression model based on current scientific literature on smoking cessation predictors, as follows: gender; educational level; number of years of smoking; time to first cigarette (i.e. the length of time after waking up until the first cigarette); number of cigarettes per day; occurrence of breathlessness; difficulty in being in non-smoking areas; most difficult cigarette to quit (i.e. the first cigarette in the morning or any other cigarette); tendency to smoke most of the day during sick leave; use of nicotine patch; presence of other smokers at home; and practice of physical activity. The variable “success” was chosen as the dependent variable. These criteria were included in order to analyze the role of ETA in smoking cessation treatment during adulthood, after adjustment for possible confounders.

## RESULTS

Chi-square tests showed significant differences between the ETA and non-ETA groups in relation to the following variables (Table 1): gender (P < 0.01), occurrence of breathlessness (P < 0.05), number of years of smoking (P < 0.01), age at time of first cigarette (P < 0.01), tendency to smoke most of the day during sick leave (P < 0.01), presence of other smokers at home (P < 0.05) and practice of physical activity (P < 0.01). ETA was reported by 81% of the men and 67% of the women; 76% of those presenting breathlessness; 60% of those who had been smoking for less than 30 years; 77% of those who had been smokers for at least 30 years; 77% of those who had their first cigarette five minutes after waking up; 77% of those who lived with another smoker; and 78% of those who did not practice any physical activity. Success was associated with the non-ETA group (P < 0.05), such that 46.6% of this group were successful, versus 33.7% in the ETA group.

**Table 1.** Descriptive analysis on experimentation with tobacco during adolescence (ETA) and non-ETA groups among 367 smokers who were attending outpatient smoking cessation treatment at a psychosocial care unit (CAPS) in São Caetano, São Paulo, Brazil, 2007-2010

Variable	Non-ETA		ETA		χ^2^	P
	n	%	n	%		
Gender						
Female	79	32.6	136	67.4	7.38	0.007
Male	24	19.2	101	80.8		
Educational attainment (years of schooling)						
Up to 8 years	45	33.8	88	66.2	3.44	0.064
9 or more years	58	24.8	176	75.2		
Breathlessness						
No	49	34.3	94	65.7	4.46	0.035
Yes	54	24.1	170	75.9		
Number of years smoking						
< 30	46	39.66	70	60.34	11.2847	0.001
≥ 30	57	22.71	194	77.29		
Cigarettes per day						
< 30	71	31.7	153	68.3	3.7543	0.053
≥ 30	32	22.38	111	77.62		
Time to first cigarette in the morning						
≤ 5 minutes	50	22.83	169	77.17	7.3699	0.007
> 5 minutes	53	35.81	95	64.19		
Difficulty staying in non-smoking areas						
No	51	32.,9	104	67.1	3.0139	0.083
Yes	52	24.64	159	75.36		
Most difficult cigarette to quit						
First cigarette in the morning	70	29.29	169	70.71	0.6738	0.412
Any other cigarette	31	25.2	92	74.8		
Tendency to smoke during a sick period						
No	50	41.32	71	58.68	15.1435	< 0.01
Yes	52	21.76	187	78.24		
Other smokers at home						
No	56	34.36	107	65.64	5.6111	0.018
Yes	47	23.15	156	76.85		
Physical activity						
No	46	22.22	161	77.78	7.3075	0.007
Yes	55	35.03	102	64.97		
Nicotine patch						
No	4	18.18	18	81.82	1.1483	0.284
Yes	99	28.78	245	71.22		
Bupropion						
No	55	28.35	139	71.65	0.0166	0.898
Yes	48	27.75	125	72.25		
Nortriptyline						
No	100	28.99	245	71.01	2.1152	0.146
Yes	3	14.29	18	85.71		
Nicotine gum						
No	85	29.41	204	70.59	1.0952	0.295
Yes	18	23.38	59	76.62		
Success						
No	55	23.91	175	76.09	5.622	0.022
Yes	48	35.04	89	64.96		
Total	103	28.1	264	71.9		

Table 2 presents the results from the crude and adjusted logistic regression models for treatment success. As shown, ETA was significantly associated with failure of the treatment (odds ratio = 0.57; 95% confidence interval = 0.33-0.96; P < 0.05). A longer time until the first cigarette and a greater number of years of smoking (duration of smoking) were associated with success. In further crude analysis, duration of smoking (both continuous and categorical) was associated with success. Male gender, low education level, hypertension and shorter time until the first cigarette were associated with the class with greater number of years of smoking, in the adjusted analysis. No multicollinearity was found, i.e. the variance inflation factor (VIF) values were very low. No significant difference was found between the ETA and non-ETA groups regarding treatment drop-out rate (55.9% versus 48.5%, respectively). A further crude and adjusted logistic regression was carried out excluding those who dropped out. Success continued to be associated with the non-ETA group (P < 0.05), which had a success rate of 90.6%, versus 76.7% in the ETA group.

**Table 2.** Results from multiple logistic regression model regarding success in smoking cessation among 367 smokers who were attending outpatient smoking cessation treatment at a psychosocial care unit (CAPS) in São Caetano, São Paulo, Brazil, 2007-2010

Variables	n	%	OR	aOR	[95%	CI]	P	VIF
Exposure								
Non-ETA	48	46.6	1.00	1.00				
ETA	89	33.7	0.58	0.57	0.33	0.96	0.037	1.17
Covariates								
Gender								
Female	96	39.7	1.00	1.00				
Male	41	32.8	0.74	0.77	0.45	1.29	0.324	1.17
Education level								
Up to 8 years	58	43.6	1.00	1.00				
9 or more years	79	33.8	0.65	0.68	0.42	1.09	0.108	1.05
Number of years smoking								
Less than 30 years	34	29.3	1.00	1.00				
30 years or more	103	41.0	1.67	1.93	1.15	3.25	0.014	1.08
Time to first cigarette in the morning								
Up to 5 minutes	69	31.5	1.00	1.00				
More than 5 minutes	68	46.0	1.84	1.76	1.07	2.88	0.027	1.20
Cigarettes per day								
Less than 30	91	40.6	1.00	1.00				
30 or more	46	32.2	0.69	0.92	0.55	1.54	0.748	1.22
Breathlessness								
No	50	35.0	1.00	1.00				
Yes	87	38.8	1.18	1.57	0.95	2.60	0.077	1.13
Difficulty being in non-smoking areas								
No	64	41.3	1.00	1.00				
Yes	72	34.1	0.73	0.81	0.49	1.35	0.425	1.26
Most difficult cigarette to quit								
First cigarette in the morning	100	41.8	1.00	1.00				
Any other cigarette	36	29.3	0.57	0.65	0.39	1.06	0.090	1.05
Tendency to smoke most of the day during sick leave								
No	51	42.2	1.00	1.00				
Yes	84	35.2	0.74	0.83	0.48	1.42	0.497	1.31
Nicotine patch								
No	4	18.2	1.00	1.00				
Yes	133	38.7	2.83	2.42	0.72	8.08	0.152	1.10
Other smokers at home								
No	58	35.6	1.00	1.00				
Yes	79	38.9	1.15	1.16	0.72	1.86	0.535	1.08
Physical activity								
No	75	36.2	1.00	1.00				
Yes	62	39.5	1.14	1.08	0.67	1.75	0.755	1.13

Non-success was the reference category; aOR = adjusted odds ratio; CI = confidence interval; VIF = variance inflation factor; ETA = experimentation with tobacco during adolescence.

## DISCUSSION

Our study shows that experimentation with tobacco during adolescence is an influential factor regarding failure of smoking cessation, independently of the level of dependence, number of years of smoking and other potential confounders. Consistent with the available literature,^14^^,^^22^ our results confirmed that experimentation with tobacco during adolescence was also associated with greater severity of smoking history, such as having been a smoker for longer and reporting occurrences of the symptom of breathlessness. Since the risk of developing tobacco-related disease increases as a function of the duration of time for which tobacco is used, adolescent users are at particularly high risk of physical consequences from tobacco use later on.

Tobacco smoking among adolescents remains a persistent threat to public health,^1^ and most adolescents who use tobacco regularly (e.g. monthly or more frequently) continue their use into adulthood. For example, while only 5% of adolescent smokers see themselves as continuing to smoke five years later, 75% are actually still smoking eight years later.^23^

Defining the boundaries of this period and what it encompasses is a matter of controversy. A cross-sectional study revealed differences in brain activation between late adolescents (18-19 years old) and young adults (23-25 years old) during cognitive control, thus indicating that protracted functional development of the cortex continues into these individuals’ twenties.^24^ Moreover, maturational changes in active synaptic pruning, a process that is thought to enhance information processing capacity and speed and information rearrangement, and in white matter myelination, a process that aids the functional integration of widely distributed circuitry, begin to accelerate during early adolescence and reach a plateau approximately at the ages of 24-25 years.^25^

It is well known that adolescence is a crucial period for the development of cognitive and executive functions, working memory, reward processing, emotional regulation and motivated behavior. There is greater vulnerability to the effects of nicotine during this period. More specifically, the neurobiological impact of early-onset smoking may be especially deleterious because maturational changes in active synaptic pruning and rearrangement and white matter myelination begin to accelerate during early adolescence.^26^ Persistent exposure to nicotine via tobacco smoking throughout adolescent neurodevelopment can damage newly maturing synaptic signaling pathways and alter the patterns of neurotransmitter release, thereby increasing sensitivity to reward-related neural activation and susceptibility towards developing nicotine dependence.^27^ One recent laboratory study^28^ demonstrated that cigarette craving, as assessed through the statement “Nothing would be better than smoking a cigarette right now”, was greater among early-onset smokers than it was among late-onset smokers and healthy non-smokers. Through use of electroencephalography and event-related potentials, this study^28^ showed elevated reactivity in the early-onset group after these individuals viewed salient smoking-related images.

Positive expectancies develop as a result of heightened sensitivity to rewards during this period and often when there is a pleasurable initial smoking experience. However, there are also particular risk factors to be addressed regarding the easy availability of cigarettes and the positive social norms regarding smoking.^13^ The extent to which cigarette availability (perhaps concomitant to exposure to peers) affects reward-related processing among adolescent smokers has not been fully examined. This remains an important target for future research. Nonetheless, the findings of the present study add strength to policies that aim to restrict adolescents’ access to tobacco.

The finding that greater numbers of years of smoking was associated with success in the treatment was conflictive with some opposite findings in the recent literature.^29^ However, it is important to note that the evidence from the previous study came from a general population sample, which differed from the type of sample of the present study (clinical). In addition, more than 70% of the subjects included in the present study were undergoing psychiatric treatment for mental disorders other than nicotine dependence. Such populations are known to have exceptionally high smoking rates, due to biological, psychological and social factors. Moreover, adding to previous studies that demonstrated how starting to smoke early on acts as a negative predictor for cessation success,^9^^,^^12^ the present study also reaffirmed the importance of this variable in the specific population of individuals with mental health and addiction disorders.

The present study had limitations, especially with regard to subjective measurement of success, given that the majority of the variables were based on the patients’ self-reports. Objective data, such as quantification of salivary cotinine and carbon monoxide, could have been useful.^30^ Despite the evidence that self-reported data regarding cessation of tobacco use is reliable,^29^ biological data such as assessment of the quantities of salivary cotinine and carbon monoxide could have been useful. However, there is evidence showing that self-reported data on cessation of tobacco use is reliable.^31^ Presentation of severe unpleasant withdrawal symptoms during the first days after quitting and the consequent higher likelihood of relapse emphasize the relevance of maintaining abstinence during this period in order to achieve long-term cessation. In middle-income countries, studies conducted on anti-smoking treatment in community settings have lacked instruments to objectively quantify nicotine abstinence and, thus, have continued to use self-report measurements.^10^^,^^32^^,^^33^^,^^34^ Others limitations that need to be added were that the number of patients excluded was not recorded according to cause and that there was a high drop-out rate.

Our results may add clinical considerations to the neurobiological evidence available in relation to experimentation with tobacco during adolescence.^14^ One particular feature of the treatment protocol that was used at this unit was that cognitive-behavioral therapy in groups was provided, conducted by a psychologist with broad experience relating to addiction.^20^ This type of experience differs from the supportive counselling that is provided by general practitioners in most smoking cessation clinics.

## CONCLUSIONS

Early exposure to nicotine may lead to higher risk of continuing smoking after treatment in adulthood. This finding needs to be incorporated into prevention and treatment strategies, in order to enhance health literacy regarding severe modifiable risk factors within smoking.
